# Valorisation of cotton post-industrial textile waste into lactic acid: chemo-mechanical pretreatment, separate hydrolysis and fermentation using engineered yeast

**DOI:** 10.1186/s12934-024-02384-3

**Published:** 2024-04-10

**Authors:** Marta Simonetti, Pietro Butti, Raffaella Desiré Di Lorenzo, Valeria Mapelli, Paola Branduardi

**Affiliations:** 1 Cotonificio Albini S.P.A., Albino, 24021 Bergamo, Italy; 2 IndBiotech Lab, Department of Biotechnology and Biosciences, University of Milano Bicocca, Piazza Della Scienza 2, 20126 Milan, Italy

**Keywords:** Textile waste, Enzymatic hydrolysis, Yeast fermentation, Lactic acid, Circular bioeconomy

## Abstract

**Background:**

The textile industry has several negative impacts, mainly because it is based on a linear business model that depletes natural resources and produces excessive amounts of waste. Globally, about 75% of textile waste is disposed of in landfills and only 25% is reused or recycled, while less than 1% is recycled back into new garments. In this study, we explored the valorisation of cotton fabric waste from an apparel textile manufacturing company as valuable biomass to produce lactic acid, a versatile chemical building block.

**Results:**

Post-industrial cotton patches were pre-treated with the aim of developing a methodology applicable to the industrial site involved. First, a mechanical shredding machine reduced the fabric into individual fibres of maximum 35 mm in length. Afterwards, an alkaline treatment was performed, using NaOH at different concentrations, including a 16% (w/v) NaOH enriched waste stream from the mercerisation of cotton fabrics. The combination of chemo-mechanical pre-treatment and enzymatic hydrolysis led to the maximum recovery yield of 90.46 ± 3.46%, corresponding to 74.96 ± 2.76 g/L of glucose released, which represents a novel valorisation of two different side products (NaOH enriched wastewater and cotton textile waste) of the textile industry. The *Saccharomyces cerevisiae* strain CEN.PK m850, engineered for redirecting the natural alcoholic fermentation towards a homolactic fermentation, was then used to valorise the glucose-enriched hydrolysate into lactic acid. Overall, the process produced 53.04 g/L ± 0.34 of l-lactic acid, with a yield of 82.7%, being the first example of second-generation biomass valorised with this yeast strain, to the best of our knowledge. Remarkably, the fermentation performances were comparable with the ones obtained in the control medium.

**Conclusion:**

This study validates the exploitation of cotton post–industrial waste as a possible feedstock for the production of commodity chemicals in microbial cell-based biorefineries. The presented strategy demonstrates the possibility of implementing a circular bioeconomy approach in manufacturing textile industries.

**Graphical Abstract:**

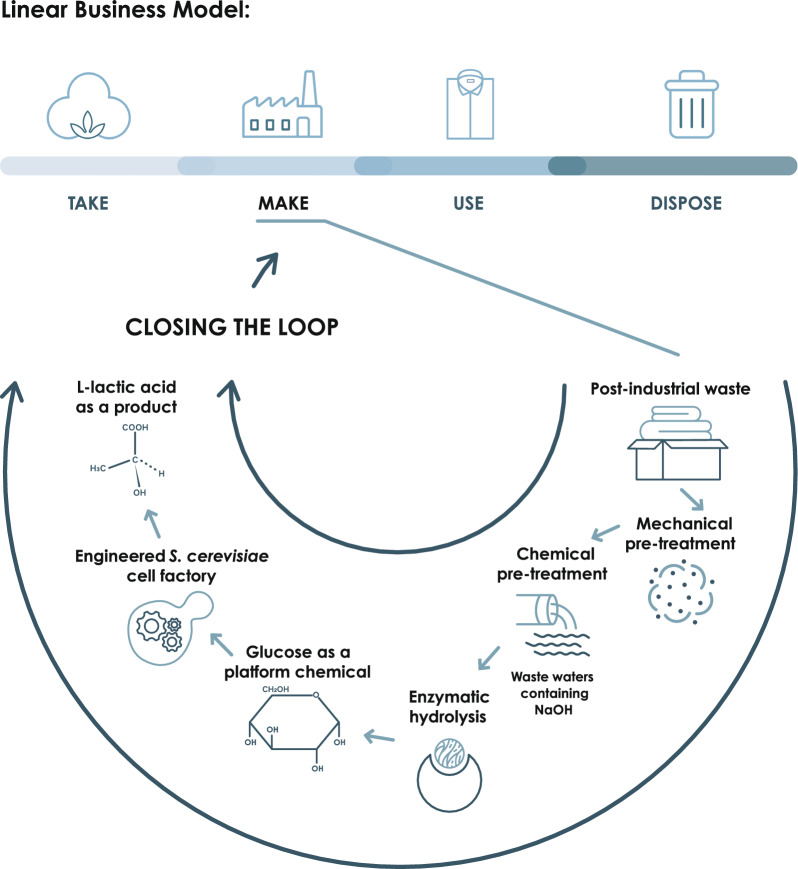

## Background

The textile industry is facing the need to adopt a more sustainable business model and turn from a linear to a circular system, prioritizing the reduction of resources and externalities. Due to the increasing need for garments, related to the growth of the population predicted up to 10 billion by 2060 [1], and to the short-term use of clothing and accessories, waste management is a major challenge for the textile industry. Globally about 75% of textile waste is disposed of in landfills, and only 25% is reused or recycled [2], while less than 1% is recycled back into new garments [3]. Cotton, a natural fibre, and polyester, a synthetic fibre, dominate the global textile market [4], therefore these two types of fibres account for the majority of textile waste [5]. The textile industry has implemented various recycling approaches to extract maximum value from waste [6], including mechanical, chemical and biological treatments that can be applied depending on the type and amount of fibres in the sorted waste and according to their origin [7]. The final purpose of recycling can be different: to regenerate new fibres and materials, or to release monomers that can be valorised in bioenergy or value-added chemicals with a biorefinery approach [8]. In particular, cotton textile waste can be considered as a valuable second-generation biomass: it is mainly constituted by cellulose (82–96%), and minor fractions of hemicelluloses (2.0–6.4%), proteins (1.0–1.9%), and other chemicals (1.2–9.6%). Waxes and pectic substances are also present, especially in the outermost layer, conferring a certain hydrophobic character [9], in an amount of 0.4–1.2%. Therefore, the high cellulose content, the negligible amount of lignin, and the restricted amount of sources of inhibitory compounds make cotton textile waste a promising source of glucose to be used in bioprocesses based on microbial cell factories, with the perspective of being competitive with other residual biomasses in terms of costs for the bioproduction of value-added chemicals [10]. With an estimated quantity of cotton textile waste exceeding 11 million tons per year [3], the development of bioprocesses based on cotton textile waste is promising in unlocking access to a highly abundant and low-cost carbon source. This, in turn, allows for a simultaneous reduction in the volumes of textile waste incinerated or landfilled.

Different studies have described that the enzymatic recovery of glucose from post-consumer textile waste can be improved by applying different combinations of mechanical and chemical pre-treatments, very likely as they increase the accessibility and the surface of contact with enzymes (Table 1).

**Table 1:** Literature comparison on enzymatic building blocks recovery from textiles

Starting material	TS (% w/v)	Pre-treatment	Hydrolysis conditions	Building blocks recovery yields	Refs
Textile post-industrial waste, 60% cotton—40% polyester	3%	Mechanical: grinding (1 × 1 cm^2^). Chemical: 7% w/v NaOH and 12% w/v urea at − 20 ℃ for 6 h. Milling with hammer crusher-powder with diameter of less than 1 mm	Cellulases (Celluclast by Novozymes) and β-glucosidase (Sunson). Citrate buffer, pH 5, 50 °C, 350 rpm, 96 h	98.3%	[11]
Purchased textile fabric, 45% wool—55% polyester, 100% polyester	2%	Mechanical: grinding (≤ 0.6 mm)	Proteases (Ronozyme ProAct) + 2% sodium thioglycolate. Two steps enzymatic hydrolysis, Tris–HCl buffer, pH 10, 50 °C, 6 h + 16 h	90–100% weight loss	[36]
Post-consumer textile waste, cotton, wool, polyester blends	1.33%	Mechanical: grinding (1 mm)	Proteases. Tris–HCl buffer, pH9, 50 °C, 400 rpm, 48 h. Cellulase cocktail. Citric buffer, pH 4.8, 50 °C, 400 rpm, 120 h	95% (wool), 85% (cellulose, 0.62 g/L of glucose)	[12]
Purchased garnments,100% cotton	5%	Mechanical: cutting, grinding (½ inch × ½ inch); milling. Chemical: ozone 3.2% and 2% w/v NaOH and 1% v/v H_2_O_2_, 70 ℃,1 h	Cellic CTec3 cellulase cocktail (Novozymes). Sodium acetate-acetic acid buffer, pH 5.2, 50 °C, 12 rpm in rotating incubator, 96 h	90%	[15]
Purchased garnments,100% cotton	5%	Mechanical: cotton grinding and mechanical refining (20,000 revs)	Cellic CTec 2 and CTec 3 cellulase cocktail (Novozymes). Sodium acetate-acetic acid buffer, pH 5.2, 50 °C, 12 rpm in rotating incubator, 96 h	89.3% (Cellic Ctec2), 98.3% (Cellic CTec 3)	[13]
Filling material for textiles, cotton and polyester blends	5%	Mechanical: cutting and grinding. Chemical: 20% w/v NaOH, 1 h, room temperature	Cellulase cocktail NS59143 (Novozymes). Citric buffer, pH 5.0, 55 °C, 48 h	99%	[33]
Purchased garnments,100% cotton, blended	1%	Mechanical: cutting. Chemical: 15% w/v NaOH; H_2_O_2_:acetic acid, 1:1; 15% w/v NaOH, 60% v/v ethanol	In-house produced RUT-30 cellulase, 45 °C, 120 rpm, 168 h	99.1%	[22]
Post-industrial cotton textile waste, 100% cotton	7.5%	Mechanical: cutting and grinding. Chemical: recycled NaOH enriched wastewater effluent	Cellulase cocktail NS59150 (Novozymes). Citric buffer, pH 5.0, 150 rpm, 55 °C, 24 h	90.46% ± 3.46 (74.96 ± 2.76 g/L of glucose)	Our work

TS, Total solid

For instance, in Li et al. [11] and Quartinello et al. [12], the application of mechanical pre-treatments on post-consumer blended textile waste before the use of cellulases allowed recovery yields of the different building blocks between 85 and 98% in 96 and120 h, respectively. Vera et al. demonstrated that combining several mechanical pre-treatments before hydrolysis with a cellulase enzyme cocktail resulted in high glucose recovery yields, from 89 to 98% in 96 h [13].

Khandaker et al. [8] reported that chemical (pre)treatments can determine efficient bioconversion thanks to the breaking of hydrogen bonds in cotton fibres, resulting in reduced crystallinity and increased amorphous region. For instance, Piribauer et al. [14] reported that alkali pre-treatment of textile waste, combined with a previous mechanical pre-treatment, leads to an increased degradation of cotton in blended cotton-polyester garments up to 83% after enzymatic hydrolysis. Also, Vera et al. demonstrated that mechanical pre-treatment in combination with chemical ozone and alkali pre-treatment resulted in glucose recovery yields of 90% after enzymatic hydrolysis [15].

The released building blocks can be in turn used as starting substrates for bioconversion using microorganisms. The main advantage of cotton as residual biomass is the possibility of obtaining highly concentrated almost pure glucose, which can be converted via microbial fermentation into virtually any bulk- or fine-chemical whose production has been described on first-generation biomass. Nevertheless, this same characteristic implies the need of supplementation of cotton-derived media with external vitamin and nitrogen sources, with an impact on the overall process costs. Some studies already explored this possibility, mainly considering the production of bulk chemicals. For instance, pre-treatment and fermentation techniques to optimize ethanol production from cotton-based pre-consumer textile waste have been extensively studied [16–21]. In particular, Cho et al. [22] proposed an integrated biorefinery process for the valorisation of textile garments, converting cotton-based, coloured cotton-based, and blended cotton-polyethylene terephthalate (PET) textile materials into value-added chemicals like bioethanol, sorbitol, lactic acid, terephthalic acid (TPA), and ethylene glycol via enzymatic hydrolysis and fermentation or hydrogenation.

Despite being very promising, all these studies are still far from a real industrial implementation, since in many cases performances are distant from being competitive with current processes. For instance, lactate was produced starting from cotton material hydrolysates using *A. succinogenes* ATCC 55618 with a yield as high as 83.67%, but a titer of 12.3 g/L, not suitable for industrial applications [22].

Our study presents an upcycling strategy for recovering post-industrial 100% cotton textile waste from Albini Group production sites: high glucose recovery both in terms of yield and titer were enzymatically obtained, and in turn glucose was microbially converted L–lactic acid at high concentrations and low pH via yeast fermentation. Lactic acid (LA) is a crucial industrial material used in various industries, including food, pharmaceuticals, cosmetics, leather, and textiles [23]. It is mostly used as a monomer of biobased and biodegradable polylactic acid (PLA) for manufacturing biodegradable plastics, which have applications in packaging, agriculture, transport, electronics, and textiles [24]. Nonetheless, LA finds application in the food industry (as pH regulator, preservative or flavouring agent), in the pharmaceutical sector (for intravascular and dialysis solutions) and in the cosmetic industry (as moisturizer, anti–acne or anti-aging) [25]. Moreover, LA is of interest as chemical feedstock for the production of propylene oxide, acetaldehyde, acrylic acid, propionic acid and ethyl lactate [25]. The global market for LA is projected to reach USD 5.80 billion by 2030, with a compound annual growth rate (CAGR) of 8.1% from 2024 to 2030 [26].

LA can be produced through chemical synthesis or microbial fermentation, offering benefits such as producing pure isomers L- ( +) or D- (−), reducing energy consumption, and using organic waste and renewable resources. 90% of lactic acid is produced through fermentation processes [27]. Lactic acid bacteria (LABs) are preferred for industrial-scale production, although they are generally not able to produce LA in the desired undissociated form, since at lower pH they show irreversible damage to metabolic functions [28].

Yeasts have been studied for their ability to tolerate low pHs and phage resilience, but they naturally ferment sugars to ethanol, instead of lactic acid. A mutant strain of *S. cerevisiae*, which is impaired in alcoholic fermentation due to deletion of pyruvate decarboxylase genes, can be used to redirect pyruvate into lactic acid by overexpressing a heterologous bacterial l-lactate dehydrogenase, allowing for large amounts of L–lactic acid production [29].

In this study we focused on the development of a process for cotton post-industrial waste hydrolysis and subsequent fermentation to the industrially relevant building block L-LA. A chemo-mechanical pre-treatment of fabrics followed by enzymatic hydrolysis was designed taking into account the minimisation of externalities and the industrial applicability from both the technical and the performances points of view, as these parameters are rarely considered in the existing studies. The glucose-enriched supernatant obtained by cotton enzymatic hydrolysis was then fermented to L-LA using an engineered yeast strain. Fermentation performances are very similar to those obtained from the control cultures, demonstrating the absence of inhibitory compounds affecting the fermentation and the possibility to use cotton textile waste hydrolysate as an abundant and low-cost carbon source for microbial bioconversions with high yields.

## Materials and methods

### Fabric samples and chemicals used for analytics and pretreatment

Samples of industrial textile 100% cotton greige waste from the weaving process were provided by Albini Group (Bergamo, Italy). FTIR-ATR in compliance with UNI EN ISO 1833-1:2020-UNI EN ISO 1833-2:2020 analysis was performed to characterize the sample collected and showed that the textile waste consisted of 100% cellulosic natural fibres. UNI EN ISO 1833-1:2020-UNI EN ISO 1833-2:2020 is a common analytical method for the quantitative chemical analysis of various mixtures of fibres in textiles [30]. The composition was carried out according to three different directives: the quantitative determination of water-soluble extract with sulfuric acid 72% (v/v) (purchased from Carlo Erba Reagents S.r.l., MI, Italy), the quantitative determination of solvent-soluble extract in compliance with UNI 9273:1988, and the determination of ash content in compliance with UNI 8047:1980. Compositional analysis was carried out on 100% cotton greige fabrics samples, with cellulose representing 92% of the composition.

Citric acid monohydrate C_6_H_10_O_8_, purchased from Sigma-Aldrich Co., St Louis, MO, USA, was used to prepare the buffer solution 50 mM at pH 5 for the enzymatic hydrolysis. Lab-grade sodium hydroxide for chemical pre-treatment was purchased from Sigma-Aldrich Co., St Louis, MO, USA, while industrial grade sodium hydroxide, used in mercerizing production processes, was purchased from Chimica Rainoldi S.P.A., BG, Italy by Albini Group.

### Mechanical and chemical pretreatments

Textile waste was subjected to two different pre-treatments before the enzymatic hydrolysis. In one case, only a mechanical pre-treatment was executed, in the second case a chemical pre-treatment was also applied.

**Fig. 1:**
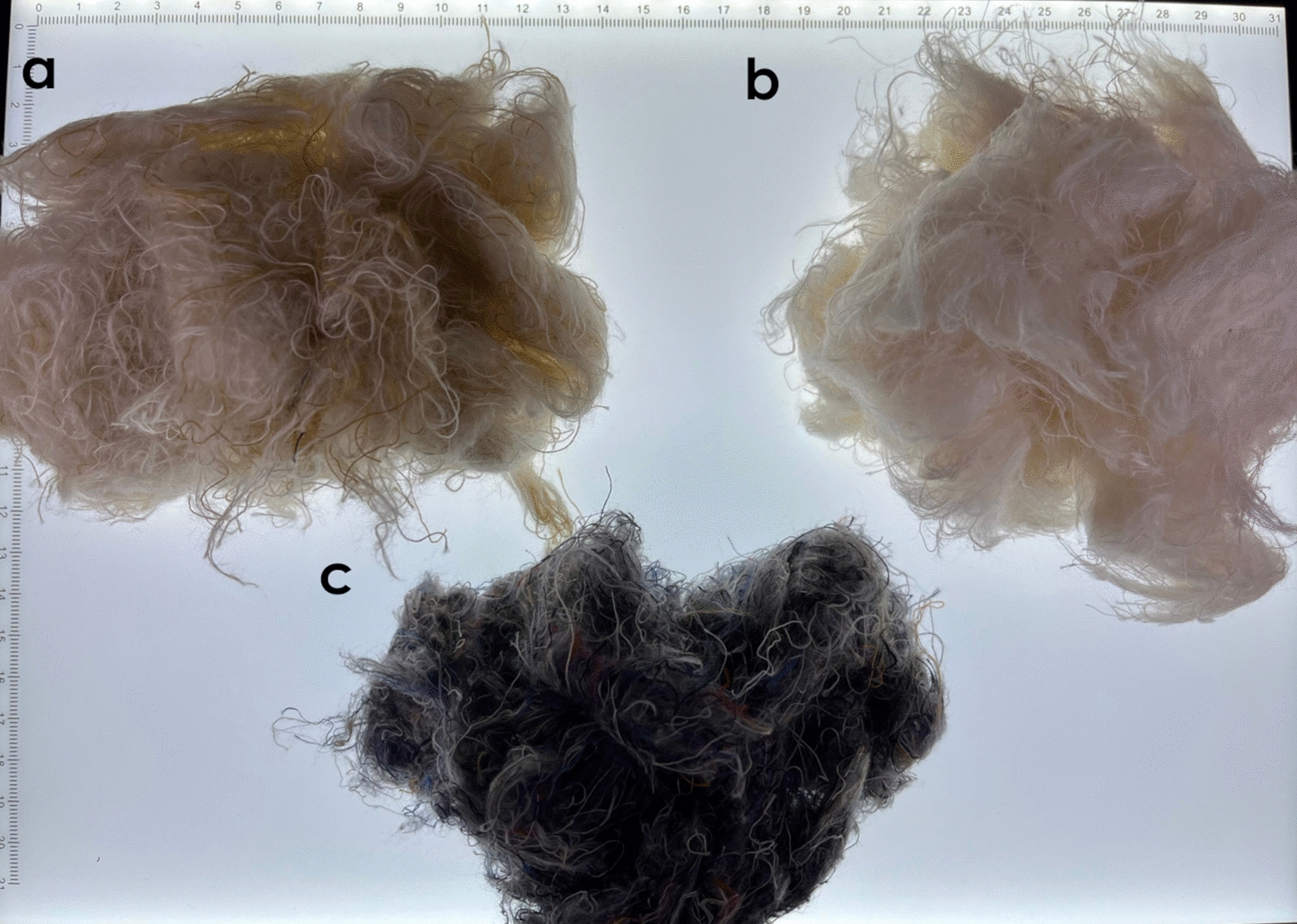
Textile waste from Cotonificio Albini mechanically treated by the supplier Cascamificio Bergamasco S.R.L. **a** sorted 100% cotton textile waste-treatment to obtain longer fibres **b** sorted 100% cotton textile waste-optimized process to reduce the dimension of the fibres **c** unsorted textile waste. Reference standard for the grid: cm

For mechanical pre-treatment, the collected fabric waste was processed on dedicated cutting lines to avoid contamination by other fibres (Fig. 1). This procedure was executed by Cascamificio Bergamasco S.R.L., using an in-house customized fraying machine. Patches moved over a floating roller, starting with the coarsest cut and progressing to the tiniest sizes, reducing cotton residues to individual fibres of varying lengths from 15 to 35 mm. The machine production capacity was 300–400 kg/h, varying on the homogeneity and absence of impurities in the material. Textile waste collected from Albini Group weaving department may contain residues such as paper, adhesive tape, plastic and dyed fabrics, for this reason, the material is manually sorted to guarantee homogeneity before mechanical pre-treatment.

For chemical pretreatment, the biomass was immersed in a solution containing laboratory-grade or industry-grade NaOH at different concentrations, 50%, 20%, 10% and 5% w/v. Moreover, wastewater recovered from the mercerization process, containing 16% (w/v) NaOH, were also used. The amount of mechanically treated cotton waste was at a weight/volume ratio of 7.5%, unless differently indicated. An operative volume of 30 mL was used during the assessment of the process conditions while it was set to 500 mL for preparative hydrolysis. The chemical pre-treatment lasted for 2 h, at room temperature, without agitation. Samples were then washed 4 times with citrate buffer 50 mM, pH 5. Subsequently, 37% (v/v) HCl was added to the samples to achieve a pH between 4.5 and 5.5 and the samples were further washed 2 times to remove NaCl derived from the neutralization process and resuspended in the same buffer to achieve the suitable pH for subsequent enzymatic hydrolysis. All the samples, comprising the ones only mechanically pre-treated, were left in a citrate buffer at room temperature overnight.

### Determination of enzymatic activity by Filter Paper Assay (FPA)

Enzymatic cocktail NS59150, a cellulases/hemicellulases blend, kindly provided by Novozymes (Novozymes A/S, Copenhagen, Denmark), was used for the hydrolysis of textile waste. Public information about the enzymatic cocktail is not available because sensitive information about the enzyme sample, such as specific components, must not be disclosed. The activity of cellulases was determined by following the NREL protocol from the National Renewable Energy Laboratory using the filter paper assay (FPA) as described by the IUPAC [31]. This test yields filter paper units (FPU) per gram or millilitre, measuring the quantity of enzyme required to hydrolyse a piece of filter paper of a standard size, used as a reference cellulosic substrate. Hydrolysis was performed with 50 mg Whatman no.1 filter paper strip in 1 ml citrate buffer 0.05 M with 0.5 ml of enzyme diluted in citrate buffer. Incubation was carried out at 50 °C for 60 min. The reaction was stopped by adding 3 ml of 3,5-dinitrosalicylic acid (DNS) reagent, then the solution was boiled, and a colour reaction took place, depending on the concentration of sugar released in the hydrolysis reaction. The change in the photo-absorbance value of this mixture was measured at 540 nm, and by comparing it to a calibration curve created by glucose standards (working stock solution 10 mg/mL), the FPU was calculated. The quantification of cellulase activity was equal to 137 FPU/mL for the Novozyme enzymatic cocktail NS59150.

### Enzymatic hydrolysis

The enzymatic hydrolysis was conducted on pre-treated biomass, either only mechanically or also chemically processed. The operative volume was set at 30 mL during the evaluation of process conditions and increased to 500 mL for preparative hydrolysis. The amount of biomass was at a weight/volume ratio of 7.5%, if not differently indicated.

Next, 100 µL/g biomass of NS59150 enzyme cocktail, equal to 13.7 FPU/g biomass, was added to the samples, which were then incubated at 150 rpm at 55 °C for 24 h, sampling the reaction over time.

The enzyme loading was decided based on literature studies, which reported the use of this amount for commercially available enzymatic cocktails. To investigate the possibility of using lower quantities of enzyme cocktail, different enzyme loads in addition to the initial set-point of 100 µL/g biomass were tested, namely: 1 μL/g biomass, 10 μL/g biomass, 50 μL/g biomass, 100 μL/g biomass. We also tested the condition of 125 μL/g biomass, to exclude the need for higher amounts for the experimental cocktail in use.

All the experiments were carried out in triplicates, except for the preparative hydrolysis for the subsequent microbial fermentation, which was performed in duplicate.

The pH of the enzymatic reaction mixture was measured at the beginning and end of the hydrolysis to verify the maintenance of the enzyme’s optimum pH value using pH indicator strips, in order to maintain sterility and minimize volume losses.

### Homolactic fermentative yeast strain used in the study

*S. cerevisiae* strain CEN.PK m850 derives from strain CEN.PK RWB876 (MATa *pdc1(–6–2)::loxP pdc5(–6, –2)::loxP pdc6(–6, –2):loxP ura3-52* YEpLpLDH) [32]. In this strain the alcoholic fermentation is impaired because of the deletion of all the pyruvate decarboxylase encoding genes. The expression of a bacterial lactate dehydrogenase allows the occurrence of a lactic fermentation. Strain CEN.PK m850 was selected after adaptive laboratory evolution for augmented tolerance to the presence of high concentration of lactic acid at pH values lower than 2.5. This strain was used for lactic acid production. It was stored at − 80 °C in 20% glycerol (v/v).

### Fermentation of textile waste hydrolysate for lactic acid production

For lactic acid production, *S. cerevisiae* CEN.PK m850 was grown as described by [29], with minor changes indicated below. In brief, the strain was plated to saturation on agar plates containing 1.7 g/L yeast nitrogen base (YNB) without amino acids, 10 g/L ethanol, and 10 g/L glycerol. Then, cells were harvested and used to inoculate 100 mL of pre-inoculum medium (0.31 g/L CaCO_3_, 1.7 g/L YNB without amino acids, (NH_4_)_2_SO_4_, 1.5 g/L urea, 10 g/L ethanol, and 0.5 g/L glucose) at an optical density at 600 nm (OD_600_) of 0.3, in 250 mL baffled shake flasks (air-to-liquid ratio 2.5:1). After 24 h of growth, the cells were harvested and used to inoculate 100 mL of fermentation medium at an OD_600_ of 3.0, in 250 mL baffled shake flasks (air-to-liquid ratio 2.5:1). The fermentation medium was obtained by supplementing the hydrolysate with 4.5 g/L CaCO_3_, 1.7 g/L YNB without amino acids and (NH_4_)_2_SO_4_, 1 g/L urea, 5 g/L ethanol. For control experiments, pure laboratory-grade glucose at the initial concentration of 65 g/L was used as a carbon source instead of the cotton hydrolysate. Citric buffer 44.683 mM was also added where indicated. Samples were collected every 12 h to determine the OD_600_, pH, glucose consumption, and lactic acid production.

### Analytical methods

The optical density of *S. cerevisiae* CEN.PK m850 (OD_600_) was measured using a Shimadzu UV-1800 spectrophotometer (Shimadzu Corporation). When CaCO_3_ was still present in the medium, the cell suspension was diluted using HCl 0.1 M to solubilize precipitates interfering with OD_600_ measurement.

Glucose, l-lactic acid, ethanol, acetic acid, and glycerol were quantified by HPLC analysis using the Rezex ROA-Organic Acid (Phenomenex) column on an Agilent 1100/1200 series. The eluent was 0.01 M H_2_SO_4_ pumped isocratically at a flow of 0.8 mL/min for 10 min with column temperature maintained at 80 °C. Separated components were detected using a refractive index detector, and peaks were identified by comparison with known reference standards. Samples were centrifuged at 14,000 g for 8 min and filtered before the analysis.

The pH was measured using indicator strips at the beginning and end of the hydrolysis.

## Results and discussion

### Pretreatment of industrial cotton waste and glucose recovery

In this study, we aimed to valorise post-industrial cotton fabrics in a logic of closing the loop, since, in terms of circular economy perspective, the global fashion industry is interested in reducing externalities, among which textile waste constitutes a relevant fraction. Considering the high-quality standards of the fabric produced, the possibility of recycling the material to re-obtain fabrics is not an option. As an alternative, we considered enzymatic hydrolysis and microbial fermentation for the valorisation of cotton as a residual biomass. We opted for using 100% cotton greige textile scraps derived from the weaving process as representative of cotton pre-consumer waste. In order to evaluate the efficacy of the following procedures, it was primarily necessary to quantify the cellulose content of the used cotton fabric samples, as reported in “Materials and Methods” section. From the analysis, it resulted that the cellulose represents 92% of the total material.

As reported in the introduction, a combination of mechanical and chemical pre-treatment can be the optimal condition for favouring the enzymatic hydrolysis. A first step of mechanical pre-treatment was applied using a textile recycling machinery, obtaining fibres of a length between 15 and 35 mm. Furthermore, alkaline treatment was considered to favour the transition of cellulose from the crystalline to the amorphous state, as suggested in previous studies [8]. Since sodium hydroxide is a widely used chemical in the textile industry in mercerizing procedures as pre-treatment of yarns and fabrics, another side-stream, constituted of alkaline wastewater, has been tested as a possibility to further reduce the need for virgin reagents in the developed valorisation process.

**Fig. 2:**
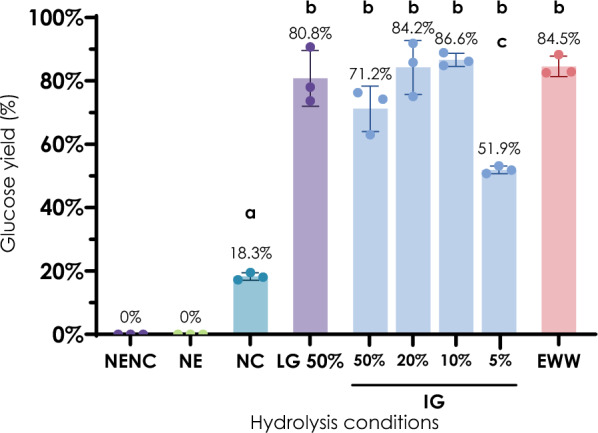
Glucose yields obtained with different pretreatment conditions of cotton waste material. NENC: No Enzymes No Chemical pretreatment control, mechanically treated sample exposed to the same buffers and solutions, without chemical and enzymatic treatments. NE: No Enzymes control, mechanically and chemically pretreated sample without the addition of enzymes. NC: No Chemical pretreatment control, only mechanically pretreated sample with the addition of enzymes. LG: mechanical and chemical pretreatments, with Lab Grade NaOH and hydrolysis. IG: mechanical and chemical pretreatments with Industrial Grade NaOH and hydrolysis. EWW: mechanical and chemical pretreatments with Effluent Wastewater containing NaOH. **a** p < 0.05 compared to NENC, **b** p < 0.0001 compared to NC, **c** p < 0.0001 compared to IG 20% and IG10%

To identify ideal working conditions and to verify the suitability of this effluent for chemical pre-treatment, different concentrations of the industrial-grade sodium hydroxide used in the mercerization plant (50%, 20%, 10%, and 5%) were compared, using lab-grade 50% NaOH and a non-treated sample as positive and negative controls respectively. In addition, the effluent of mercerizing, containing 16% NaOH was considered to verify its suitability as a substitute for pure NaOH (Fig. 2). For this set of tests, the amount of biomass used was 5.33% w/v in a final volume of 30 ml. The pre-treatment was conducted for 2 h at room temperature, without shaking as reported in literature studies [14, 33]. To reach a pH 5 the samples were washed and then resuspended in citrate buffer 50 mM overnight.

After the pre-treatment, the biomass has been incubated with the cellulase cocktail NS59150 for enzymatic hydrolysis of cellulose to glucose, with an enzyme load of 100 μL NS59150/g of cotton biomass (corresponding to 13.7 FPU/g biomass). According to the manufacturer's indication, the optimal temperature and pH (55 °C and pH 5) were used, and samples were taken after 24 h of incubation for glucose measurement. Mechanically pretreated samples with and without a chemical pre-treatment with 50% NaOH were incubated without the addition of the enzymatic cocktail, as negative controls.

Figure 2 reports the yields of hydrolysis at 24 h, calculated on the total cellulose content and normalised for the volume subtracted for sampling over time. The negative controls confirm that no glucose is released as a consequence of incubation in the buffer and of the alkaline treatment alone, confirming that the glucose release is completely related to enzymatic hydrolysis.

Furthermore, it is possible to notice that when enzymatic hydrolysis is applied to the samples only mechanically pre-treated (NC), the glucose recovery is very low, attesting the necessity of a chemical pre-treatment to make cellulose fibres more accessible to enzymes.

Considering the samples after alkaline pre-treatment, it is possible to assess that NaOH concentrations above 10% allow a glucose recovery of around 80%. A further increase in the concentration does not correspond to an increase in the yield, while the glucose recovery drastically drops at lower concentrations. Remarkably the wastewater effluent enriched with 16% NaOH has a concentration above this threshold and is still as effective as the industrial- and lab-grade reagents.

These results demonstrated the possibility to exploit directly the wastewaters deriving from the mercerizing process at the Albini Group’s finishing site for the chemical pre-treatment of cotton waste, further reducing the environmental impact of the up-cycling process.

### Evaluation of hydrolysis conditions

Based on the preliminary data, further experiments were performed testing different enzyme loads as first, and afterwards evaluating cotton textile waste total solids (TS) up to the technically feasible maximum (10% w/v).

**Fig. 3:**
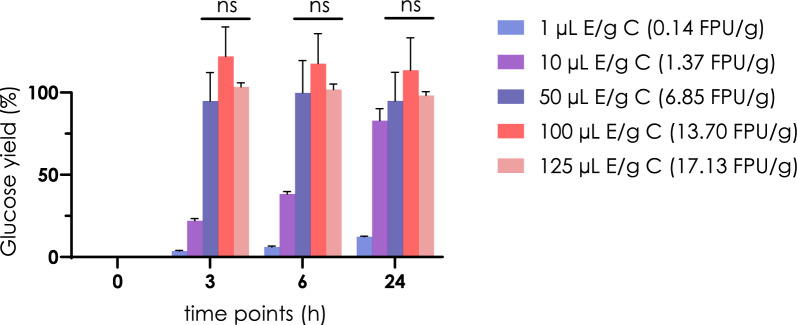
Effect of different enzyme loads on glucose yield. The glucose release yield was measured over time for different quantities of enzymes on samples mechanically and chemically pretreated, with a TS of 0.5% μL E/g C μL of enzymatic cocktail per gram of cotton residual biomass. ns, statistically not significant

Different loads of Novozyme enzymatic cocktail NS59150 were investigated on a TS of 0.5%: 100 µL/g of biomass, was kept as a reference and then we selected 3 lower concentrations, respectively 1, 10, 50 μL/g of biomass, and one higher, 125 µL/g of biomass, to verify if a higher amount of enzymes can have a benefit on the glucose recovery. The TS was kept lower than previous experiments, to avoid product inhibition. Samples were incubated as before and glucose yield was calculated at different time points, until 24 h (Fig. 3).

According to these tests, the minimal enzyme load sufficient to reach yields of glucose around 100% was 50 µL of enzyme/g of biomass, while lower enzyme loading resulted in lower yields. Although lower enzyme loads presented an increased yield in glucose recovery over time, we decided to not prolong the incubation time after 24 h, as this would anyway negatively affect the overall cost of an industrial process. Differently, it remains to evaluate if it is industrially more convenient in terms of cost and allocated time to keep the highest enzyme loading and reduce incubation time, or the opposite. Importantly, we could assess that there is no advantage in loading higher amounts of enzyme cocktail.

**Fig. 4:**
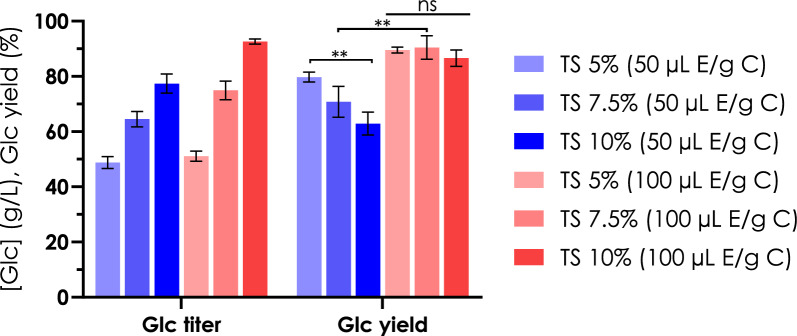
Comparison of different TS and enzyme loads. Glucose titers and yields at 24 h are shown to compare the performance of different combinations of enzyme loads (50 and 100 µL enzyme/g cotton biomass, respectively in blue and red) and TS amounts (5, 7.5, and 10%, different shades) tested during process evaluation experiments. TS: Total Solid. μL E/g C: microliters of enzyme cocktail per gram of cotton biomass. **p < 0.05 comparing TS 5% and 10% with 50 µL enzyme/g of cotton, as well as comparing TS 7.5% with 50 and 100 µL enzyme/g of biomass. ns: not significant

In the second set of experiments, we tested the combinations of enzyme loads of 50 and 100 µL/g of cotton with increasing the TS amount from 5, to 7.5, and 10% (Fig. 4).

As expected, increasing the biomass leads to the increase of the sugar release, independently from the enzyme load. Nonetheless, while with the lower biomass load after 24 h the recovery of sugar is pretty similar using an enzyme load of 50 or 100 µL/g of biomass, when moving to 7.5% and 10% TS it is necessary to use more enzyme for reaching the performances expected, probably due to product inhibition.

According to the comparison between different hydrolysis conditions and the processability of the cotton waste, the combination of enzyme load of 100 µL/g biomass (corresponding to 13.7 FPU/g biomass) and TS amount of 7.5% was judged as the most suitable for the subsequent steps: in these conditions, we obtained a reproducible recovery yield of 90.46 ± 3.46%, corresponding to 74.96 ± 2.76 g/L of released glucose.

### Production of l-lactic acid from cotton waste

Having established a suitable protocol for obtaining industrial cotton waste hydrolysate, the production of lactic acid was investigated, as a proof of concept of sequential fermentation of glucose released by cotton hydrolysis. Lactic acid can be produced both with chemical synthesis and fermentation, however microbial fermentation is preferred since it allows to obtain pure L ( +)- or D ( −)-LA enantiomers, instead of a racemic mixture, with the latter being typical of the chemical synthesis. This process also reduces environmental impacts and production costs [24]. The industrial process is mainly based on the exploitation of lactobacilli, which are natural producers of lactic acid, but as they do not tolerate low pH, the final product is indeed lactate, which for many applications is not the desirable form of the product.

In this work we use the previously developed engineered *S. cerevisiae* strain CEN.PK m850 [32], in which the alcoholic fermentation was fully replaced by lactic fermentation through pyruvate decarboxylases (PDC) deletion and lactic dehydrogenase (LDH) expression on an episomal plasmid. Furthermore, this strain is of particular interest for the industrial lactic acid production at low pH. In fact, the undissociated form of the acid is desired in the industrial context, as it can be used directly as preservative, or as monomer to be polymerised into poly-lactic acid, without additional downstream costs. Nonetheless, the accumulation of the product in this form can compromise yeast viability: undissociated organic acids can permeate the plasma membrane, entering in the cytoplasm where they dissociate causing intracellular acidification, in turn impairing fermentation performances and economic feasibility of the process [34]. The strain CEN.PK m850 derives from a parental strain engineered to substitute the natural alcoholic fermentation with the lactic fermentation, and then subjected to adaptive laboratory evolution for selecting variants with superior performances in terms of growth and production of L-LA at pH below 3.0. This strain is able to completely convert up to 70 g/L of glucose in less than 60 h, with very high yields and a positive impact on process performances [32].

A separate hydrolysis and fermentation (SHF) strategy was chosen to allow to match the optimal temperature of both the enzymatic cocktail and the microorganism (55 °C and 30 °C, respectively), since preliminary experiments have shown that NS59150 performances are greatly impaired at 30 °C, thus excluding a simultaneous saccharification and fermentation (SSF) setup.

For this experiment, preparative hydrolysis was performed in duplicate on a larger scale (500 mL), applying the final conditions described before. The hydrolysate glucose content after 24 h of incubation was 67.89 ± 1.44 g/L. Residual solids were removed by centrifugation and the supernatant was collected to be used as a carbon and energy source in the growth medium.

**Fig. 5:**
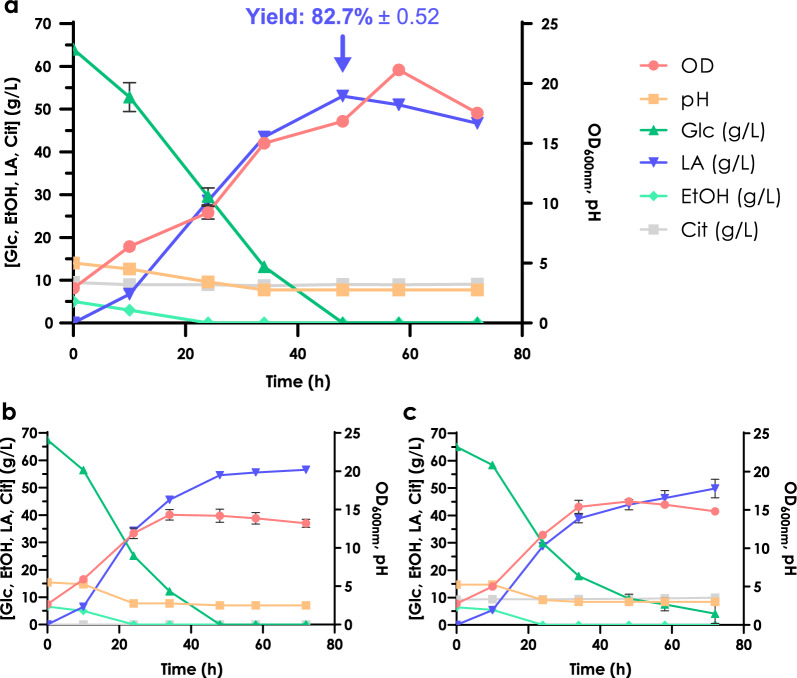
**a** l-Lactic acid production performed with cotton hydrolysate in citrate buffer and further initially buffered with CaCO3. l-Lactic acid production was performed in standard conditions as a control using both **b** lab-grade glucose + CaCO_3_ and using **c** lab-grade glucose + citrate buffer and CaCO_3_

The fermentation for lactic acid production with CEN.PK m850 strain was carried out in the standard production medium described in [29], replacing the carbon source with the hydrolysate obtained from cotton waste. In addition to the hydrolysate as the main carbon source, YNB as a source of vitamins and oligo-elements, and urea as a nitrogen source, the media is also supplemented with ethanol to allow the production of cytosolic acetyl-CoA in this PDC negative strain [35]. As this is the first time the engineered CEN.PK m850 *S. cerevisiae* strain was tested on real hydrolysates from second generation biomasses, in parallel, two control growth and production kinetics were carried out in a production medium in which pure lab-grade glucose was added at the same concentration as the one measured in the hydrolysate-containing medium, with or without the addition of citrate buffer at the concentration expected (44.69 mM). For all the fermentations, the pH of the medium was initially buffered using CaCO_3_. Main extracellular metabolites, optical density, and pH were measured every 12 h, for 72 h (Fig. 5).

**Fig. 6:**
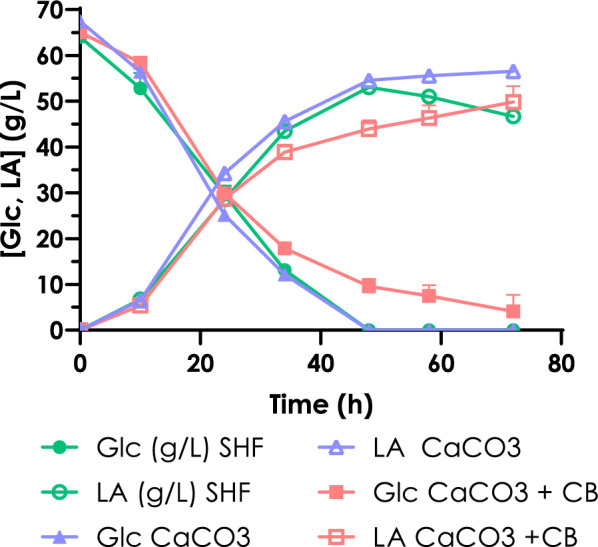
Residual glucose and l-lactic acid production kinetics for SHF with cotton hydrolysate (green), for SHF in standard conditions with laboratory grade glucose + CaCO_3_, for SHF in standard conditions with lab. Grade glucose + citrate buffer + CaCO_3_

The fermentation profile on cotton hydrolysate is overall comparable with those of control fermentations, except for an unexpected slower consumption of glucose when the reference media was supplemented with the citrate buffer (Fig. 6). Further explanations for this phenomenon are still to be investigated. Neither acetic acid nor glycerol, two common extracellular metabolites of *S. cerevisiae*, were observed in the medium, suggesting that the carbon source was efficiently converted mainly into l-lactic acid, and biomass.

Overall, a peak of production of 53.04 g/L ± 0.34 of l-lactic acid and a maximum yield of 82.72 ± 0.52% were obtained from the conversion of cotton-derived glucose into lactic acid, after 48 h of fermentation and in correspondence to a complete carbon sources depletion.

## Conclusion and future perspectives

With this study, we demonstrated the possibility of applying biochemical conversion to industrial textile waste as an alternative to current recycling procedures, with the goal of upcycling it into virgin products. A maximum sugar recovery yield of 90.46 ± 3.46% was reached, corresponding to 74.96 ± 2.76 g/L of glucose in 24 h, being the highest reported in literature from textile waste hydrolysis. The possibility of microbial bioconversion of the released glucose was then assessed, utilising directly the soluble fraction of the hydrolysate and reaching 53.04 g/L of l-lactic acid at a final pH below 3, with a yield of 82.72 ± 0.52%. Interestingly, the overall yield of the double conversion (from cotton textile waste to glucose and from glucose to lactic acid) was 75%, giving for the first time a numeric quantification of the potential of this biomass in biomanufacturing.

Furthermore, the possibility of using mercerizing wastewater as an alternative to the widely used pure NaOH was successfully evaluated for the first time, allowing to further reduce the externalities by valorising an additional waste-stream of the textile industry.

At the best of our knowledge, studies considering the possibility to exploit textile waste as a second-generation biomass are limited and this is the first report of the use of real waste from the textile industry in combination with a different side-stream used for the chemical pre-treatment, and utilizing the highest quantity of starting biomass (7.5% (w/v)), close to the maximum physically tractable [11, 13, 15, 22, 31].

Overall, this study reduces the gap to be filled for a successful industrial exploitation of low cost and highly abundant textile waste as an alternative to traditional carbon sources for microbial biomanufacturing. Considering the promising results obtained, further studies to scale up to larger volume the textile waste upcycling strategy will be held. Moreover, different microbial cell factories will be investigated to produce other valuable chemicals, with a prominent attention for those having application in the textile industry.

The bioconversion of textile waste into l-lactic acid is a promising path for industrial applications due to its dual benefits of waste reduction and valuable product generation. However, there are several challenges to be addressed. Technologically, the process requires advanced pre-treatment and conversion processes that must be optimized for efficiency and compatibility with various textile materials, particularly unsorted and mixed composition materials. Integrating these processes into existing industrial frameworks allows the development of adaptable systems that can coexist with current production methods. Economically, the viability of the bioconversion process depends on the balance between treatment and conversion costs and the market value of lactic acid. Sustainability is influenced by factors such as fluctuating feedstock prices, operating costs, and the competitive landscape of lactic acid production. Scalability is another important consideration, as the transition to industrial-scale production requires careful planning and execution, including expansion of physical infrastructure and refinement of process parameters. A holistic approach considering logistical, regulatory, and environmental issues is also necessary for successful integration. In conclusion, while the bioconversion of textile waste to lactic acid is a sustainable and economically promising way forward, the identified challenges must be addressed with innovative solutions. Future research should focus on developing more efficient pre-treatment and conversion technologies, conducting comprehensive life cycle cost assessment and life cycle assessment analyses, and developing scalable operating strategies compatible with existing industrial processes.

## Data Availability

The datasets generated and/or analysed during the current study are not publicly available because they are also part of the property of Cotonificio Albini S.p.A., a private entity, but are available from the corresponding author on reasonable request.

## References

[1] Worldometer Worldometer—real time world statistics. 2023. http://www.worldometers.info/. Accessed 25 Oct 2023.

[2] Juanga-Labayen JP, Labayen IV, Yuan Q (2022). A review on textile recycling practices and challenges. Textiles.

[3] Ellen MacArthur Foundation (2017). A new textiles economy: redesigning fashion’s future.

[4] Piribauer B, Bartl A (2019). Textile recycling processes, state of the art and current developments: a mini review. Waste Manag Res.

[5] Ütebay B, Çelik P, Çay A, Ütebay B, Çelik P, Çay A, Körlü A (2020). Textile wastes: status and perspectives. Waste in textile and leather sectors.

[6] Oliveira Silva WD, Morais DC (2022). Impacts and insights of circular business models’ outsourcing decisions on textile and fashion waste management: a multi-criteria decision model for sorting circular strategies. J Clean Prod.

[7] Lu L, Fan W, Meng X, Xue L, Ge S, Wang C (2023). Current recycling strategies and high-value utilization of waste cotton. Sci Total Environ.

[8] Khandaker S, Bashar MM, Islam A, Hossain MdT, Teo SH, Awual MdR (2022). Sustainable energy generation from textile biowaste and its challenges: a comprehensive review. Renew Sustain Energy Rev.

[9] Dochia M, Sirghie C, Kozłowski RM, Roskwitalski Z, Kozłowski RM (2012). Cotton fibres. Handbook of natural fibres.

[10] Tan J, Abdel-Rahman MA, Sonomoto K, Di Lorenzo ML, Androsch R (2018). Biorefinery-based lactic acid fermentation: microbial production of pure monomer product. Synthesis, structure and properties of poly(lactic acid).

[11] Li X, Hu Y, Du C, Lin CSK (2019). Recovery of glucose and polyester from textile waste by enzymatic hydrolysis. Waste Biomass Valoriz.

[12] Quartinello F, Vecchiato S, Weinberger S, Kremenser K, Skopek L, Pellis A (2018). Highly selective enzymatic recovery of building blocks fromwool-cotton-polyester textile waste blends. Polymers.

[13] Vera RE, Suarez A, Zambrano F, Marquez R, Bedard J, Vivas KA (2023). Upcycling cotton textile waste into bio-based building blocks through an environmentally friendly and high-yield conversion process. Resour Conserv Recycl.

[14] Piribauer B, Jenull-Halver U, Quartinello F, Ipsmiller W, Laminger T, Koch D (2020). Tex2mat—next level textile recycling with biocatalysts. Detritus.

[15] Vera RE, Zambrano F, Marquez R, Vivas KA, Forfora N, Bedard J (2023). Environmentally friendly oxidation pretreatments to produce sugar-based building blocks from dyed textile wastes via enzymatic hydrolysis. Chem Eng J.

[16] Agblevor FA, Batz S, Trumbo J (2003). Composition and ethanol production potential of cotton gin residues. Appl Biochem Biotechnol.

[17] McIntosh S, Vancov T, Palmer J, Morris S (2014). Ethanol production from cotton gin trash using optimised dilute acid pretreatment and whole slurry fermentation processes. Bioresour Technol.

[18] Jeihanipour A, Taherzadeh MJ (2009). Ethanol production from cotton-based waste textiles. Bioresour Technol.

[19] Jeihanipour A, Karimi K, Niklasson C, Taherzadeh MJ (2010). A novel process for ethanol or biogas production from cellulose in blended-fibers waste textiles. Waste Manag.

[20] Kaur U, Oberoi HS, Bhargav VK, Sharma-Shivappa R, Dhaliwal SS (2012). Ethanol production from alkali- and ozone-treated cotton stalks using thermotolerant *Pichia kudriavzevii* HOP-1. Ind Crops Prod.

[21] Plácido J, Imam T, Capareda S (2013). Evaluation of ligninolytic enzymes, ultrasonication and liquid hot water as pretreatments for bioethanol production from cotton gin trash. Bioresour Technol.

[22] Cho EJ, Lee YG, Song Y, Kim HY, Nguyen DT, Bae HJ (2023). Converting textile waste into value-added chemicals: an integrated bio-refinery process. Environ Sci Ecotechnol.

[23] Rawoof SAA, Kumar PS, Vo DVN, Devaraj K, Mani Y, Devaraj T (2021). Production of optically pure lactic acid by microbial fermentation: a review. Environ Chem Lett.

[24] Lorenzo D, Desirè R, Serra I, Porro D, Branduardi P (2022). State of the art on the microbial production of industrially relevant organic acids. Catalysis.

[25] Ahmad A, Banat F, Taher H (2020). A review on the lactic acid fermentation from low-cost renewable materials: recent developments and challenges. Environ Technol Innov.

[26] Grand view research lactic acid market to reach $5.80Bn By 2030. CAGR: 8.1%. 2024. https://www.grandviewresearch.com/press-release/global-lactic-acid-and-poly-lactic-acid-market. Accessed 4 Jan 2024.

[27] Ajala EO, Olonade YO, Ajala MA, Akinpelu GS (2020). Lactic acid production from lignocellulose—a review of major challenges and selected solutions. ChemBioEng Rev.

[28] Abedi E, Hashemi SMB 2020. Lactic acid production—producing microorganisms and substrates sources-state of art. Heliyon. 2020 https://www.cell.com/heliyon/abstract/S2405-8440(20)31817-X. Accessed 12 October 2020. 10.1016/j.heliyon.2020.e04974 PMC7566098 33088933

[29] Valli M, Sauer M, Branduardi P, Borth N, Porro D, Mattanovich D (2006). Improvement of lactic acid production in *Saccharomyces cerevisiae* by cell sorting for high intracellular pH. Appl Environ Microbiol.

[30] UNI EN ISO 1833–1:2020 - UNI Ente Italiano di Normazione. 2024. https://store.uni.com/uni-en-iso-1833-1-2020. Accessed 26 Mar 2024.

[31] Ghose TK (1987). Measurement of cellulase activities. Pure Appl Chem.

[32] Liu CL, Lievense JC 2005. Lactic acid producing yeast. WO2005052174A2, 2005. https://patents.google.com/patent/WO2005052174A2/en/enIt.pdf. Accessed 9 Jun 2005.

[33] Piribauer B, Bartl A, Ipsmiller W (2021). Enzymatic textile recycling—best practices and outlook. Waste Manag Res.

[34] Ferraz L, Vorauer-Uhl K, Sauer M, Sousa MJ, Branduardi P (2023). Impact of ergosterol content on acetic and lactic acids toxicity to *Saccharomyces cerevisiae*. Yeast.

[35] van Rossum HM, Kozak BU, Pronk JT, van Maris AJA (2016). Engineering cytosolic acetyl-coenzyme a supply in *Saccharomyces cerevisiae*: pathway stoichiometry, free-energy conservation and redox-cofactor balancing. Metab Eng.

[36] Navone L, Moffitt K, Hansen KA, Blinco J, Payne A, Speight R (2020). Closing the textile loop: enzymatic fibre separation and recycling of wool/polyester fabric blends. Waste Manag.

